# Development of paper based colorimetric method using pigment from red dragon fruit for determination of Cu and Fe

**DOI:** 10.1038/s41598-025-98693-7

**Published:** 2025-04-19

**Authors:** Rimadani Pratiwi, Raspati Dewi Mulyaningsih, Aliya Nur Hasanah

**Affiliations:** 1 Department of Pharmaceutical Analysis and Medicinal Chemistry, Faculty of Pharmacy, Universitas Padjadjaran, Jalan Raya Bandung-Sumedang KM 21, Sumedang, 45363 Indonesia; 2 Drug Development Study Centre, Faculty of Pharmacy, Universitas Padjadjaran, Jalan Raya Bandung-Sumedang KM 21, Sumedang, 45363 Indonesia; 3 Master of Pharmacy Program, Department of Pharmaceutical Analysis and Medicinal Chemistry, Faculty of Pharmacy, Universitas Padjadjaran, Jalan Raya Bandung-Sumedang KM 21, Sumedang, 45363 Indonesia

**Keywords:** Betalain, Copper, Iron, Colorimetric detection, Paper analytical device, Analytical chemistry, Natural hazards

## Abstract

Betalain, a natural pigment found in red dragon fruit, has been proposed as a natural reagent for metal detection. In the present study, this pigment was extracted and used as a colorimetric reagent for Cu and Fe on a paper-based analytical device (PAD) and then applied to water samples. The extract was stable at 5 *±* 3 °C for 16 weeks. In a solution with a pH of 4–5, the betalain extract changed color from pink to light orange (Cu) and yellow (Fe) and was selective against Na, K, Ca, Ba, Al, Mg, Zn, Hg, Ni, and Pb. The color change was caused by a metal–betalain complex with an estimated ratio of 1:2 (Cu–betalain) and 1:9 (Fe–betalain). Betalain PAD was produced under optimal conditions using Whatman CF1 paper containing 20 µL of 100 mg/mL betalain extract at pH 4–5. This process resulted in a limit of quantification of 3.133 mg/L (Cu) and 4.736 mg/L (Fe) and a limit of detection of 1.034 mg/L (Cu) and 1.563 mg/L (Fe). Based on these findings, betalain PAD made from red dragon fruit could be a viable alternative for the on-site detection of Cu and Fe in water. Ultimately, betalain PAD is a toxic waste-free, portable, fast, and prospective on-site tool for metal detection.

## Introduction

Copper (Cu) and iron (Fe) both play roles in protein synthesis in human bodies^1^. A long term deficiency or excess of both metals in the body can cause anemia, thalassemia, Menkes syndrome, and Wilson disease^2^. The highest level of metal accumulation occurs in water media, which is then distributed and contaminates other media^3^. Metals enter the body through contaminated food, polluted air, and direct contact in various industrial sectors, including pharmaceuticals^4,5^. Maintaining Cu and Fe levels in the water is an important measure to prevent excess exposure to these metals. The U.S. Environmental Protection Agency (EPA) sets maximum levels of 1.3 mg/L and 0.3 mg/L, respectively, for Cu and Fe^6^.

Recent reliable laboratory instruments for metal analysis include atomic-absorption spectroscopy, inductively-coupled plasma, and x-ray fluorescence, which can detect metals at trace levels^7–9^. Despite their sensitivity and selectivity, these methods are relatively expensive, require a long time for analysis, and remain unreliable for on-site uses. Alternative portable detectors have been developed in various forms, such as a smartphone-assisted colorimetric detector^10^, a rhodamine-based hydrogel^11^, and a 1-(2-pyridylazo) 2-naphthol colorimetric reagent^12^. On the other hand, the use of synthetic reagents on a previously developed portable detector can cause skin, nasal, and dermal irritation, in addition to producing toxic waste^13,14^.

Natural pigments have been proposed as substitutes for synthetic reagents to reduce the use of hazardous chemicals, including curcumin^15^, betalain^16^, and anthocyanin^10^ as a part of green chemistry. Overall, betalains have received less attention than curcumin and anthocyanin, which have been extensively studied as natural colors and active substances. However, betalain may represent another viable alternative colorant^17^. Betalain, a natural pigment contained in red dragon fruit, is a secondary metabolite whose biosynthesis is limited to the order Caryophyllales^18,19^. Red dragon fruit (*Hylocereus polyrhizus*) is one of the plant sources of betalain with high levels of cultivation in Asia, especially Indonesia^20^. Betalain was formerly reported to form complexes with Cu and Fe, resulting in a noticable color change followed by a shift of the maximum wavelength in the visible spectrum^21–23^. A betalain pigment-based copper detector was previously developed into a smartphone-based sensor with a modified application for analysis in liquid form^16^. Besides leading to biased color interpretation, reagents in liquid form are less convenient for on-site uses than those in solid forms.

The color-changing properties in colorimetric sensors enable several analytes to be detected using color combinations for specific identification goals^24^. This technique underlies newly developed methods such as paper-based analytical device (PAD) detection tools^25,26^. Here, paper acts as a substrate capable of providing contrasting colors for colorimetric analysis^27^. This method is also relatively inexpensive and widely available^28^. The color change effect due to the exposure of Cu and Fe in betalain allows the pigment to be used as a metal detection sensor. To date, no PAD based on betalain–metal complexes, particularly Cu–Fe metals, has been developed, despite offering significant benefits for on-site environmental monitoring. Under this background, the present study aimed to fabricate a paper-based analytical device (PAD) using betalain pigment as a natural reagent for analyzing Cu and Fe in water. This study was carried out in several stages including the extraction and characterization of betalain pigment, optimization of the reaction conditions for the betalain pigment with Cu and Fe, the optimization of PAD with betalain reagents, and performance tests.

## Experimental

### Materials and apparatus

The materials used in this research were of analytical grade, including demineralized water 99.999% (ROFA Laboratory Center, Indonesia), glacial acetic acid *≥* 99.8% (CH_3_COOH) (Merck, Germany), ammonium hydroxide 65% (NH_4_OH) (Merck, Germany), ethanol 96% (Merck, Germany), 2-propanol 99.5% (Merck, Germany), and a thin layer chromatography cellulose-coated aluminum plate (Merck, Germany). Metal salts NaCl, KCl, CaCl_2_, BaCl_2_, FeCl_3_, AlCl_3_, MgSO_4_, CuSO_4_, ZnSO_4_, Hg(C_2_H_3_O_2_)_2_, NiSO_4_, and Pb(CH_3_COO)_2_ were purchased from Merck and Sigma-Aldrich, Germany. Samples of red dragon fruit (Hylocereus polyrhizus (F. A. C. Weber) Britton and Rose) were purchased from PT. Trisna Naga Asih, Indonesia.

The instruments used in this research included a Flame Atomic Absorption Spectrophotometer (FAAS) (Perkin Elmer AAnalyst 400), a blender (Miyako BL-152 GF, Indonesia), a chromatography chamber, double-sided tape, a universal pH indicator (MColorpHast™ Merck, Germany), Whatman no. 1 paper filter, 42 ashless filter paper, and no. 6, Whatman CHR 1 chromatography paper. CF 1 and CF 3 Whatman paper was purchased from Cytiva along with 0.5 mm inert plastic, a capillary tube, a rotary evaporator (Buchi R-100), a UV-Vis spectrophotometer (Analytik Jena Specord 200), and an analytical balance (Ohaus Pioneer™ PA214).

### Betalain extraction from red dragon fruit

Fresh red dragon fruits (Hylocereus polyrhizus) were obtained from PT, Trisna Naga Asih, and selected based on their condition (i.e., intactness and lack of rotting). The fruit was picked when 30–33 days old, with a maximum maturity level of 95%, beginning at the onset of blooming. Water was used to rinse the red dragon fruit and separate the flesh from the peel. Every 400 g of flesh was chopped into small pieces and homogenized with 160 mL of water in a blender^29^. The combination was then macerated with 96% ethanol solvent at a fruit flesh (gram): ethanol (ml) ratio of 7:8^29^ for 24 h and filtered through a cotton plug in the macerator. Next, the filtrate was collected^30–32^. The filtrate was concentrated with a vacuum using a rotary evaporator at 35 °C^32^ until a thick evaporated dragon fruit extract was obtained.

### Betalain characterization

#### Qualitative analysis with thin layer chromatography

Qualitative analysis was carried out to ensure that the pigment contained was betalain, which consisted of red–purple pigment and yellow pigment. Betalain extract was spotted on a silica gel plate (5 × 10 cm). Then, the plate was eluted in a mixture of 2-propanol, ethanol, water, and acetic acid (6:7:6:1) until it extended 7.0 cm from the spot. The plate was dried and eluted again in a mixture of 2-propanol, ethanol, water, and acetic acid (11:4:4:1)^29^. The elution results were then observed under direct sunlight.

#### Qualitative analysis with UV-visible spectrophotometry

The extract was dissolved in water until we acquired either a solution of 13 mg/mL or a solution with the optimal absorbance value of 0.2–0.8^32^. Demineralised water was used as a blank. The maximum wavelength (λmax) was set to a range of 320–900 nm^31^, with a reference λmax of 530–540 nm and 480–490 nm for betacyanin and betaxanthin, respectively^33^.

#### Stability study of the betalain extract

The extract was stored at 5 *±* 3 °C in a refrigerator for a total of 16 weeks. For every measurement, the extract was freshly diluted in water to achieve a solution of 13.6 mg/mL, as the absorbance was nearly 0.700 *±* 0.02 for each 5 mL of solution. Sample preparation was conducted for each absorbance measurement at time intervals of 30, 60, 120, 180, 240, 300, and 360 min; 24 h; 2–7 days; and 2–16 weeks.

### Metal ion sensing study

#### Metal screening test

The metal screening stage aimed to determine the selectivity of betalain against exposure to metal ions, which are characterized by color changes and decreases or increases in color intensity. We acquired an extract solution whose absorbance was equivalent to 0.7–0.8^21^, and the pH was recorded as the initial pH value. Acetic acid and ammonium hydroxide were added to the extract solution to produce pH changes ranging from 2 to 11. Then, 30 mM of Na, K, Mg, Ca, Ba, Fe, Al, Cu, Zn, Hg, Pb, and Ni were added separately at a volume ratio of 1:1 for each pH level^16,21^. At a pH variation of 4–5 (initial value), no acid or base was added. To modify the pH without adding metals, the blank contained the extract, acetic acid, and ammonium hydroxide. The color change was instantly noticeable, and the absorbance was measured in scanning mode. During the screening stage, specific metals and pH values were collected, resulting in the most significant color. If any precipitation occurred in the sample, centrifugation at 5000 rpm was carried out for 5 min before spectrophotometric analysis.

#### Design and optimization of betalain paper analytical device

**Fig. 1:**
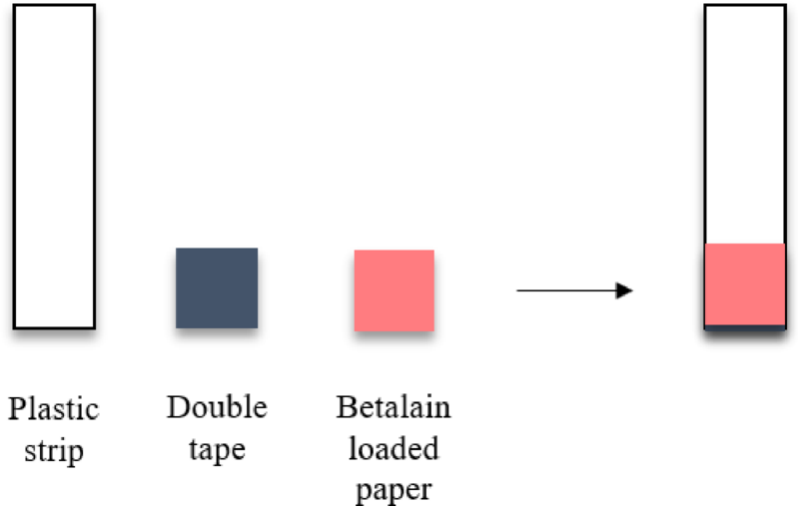
Design of the betalain paper analytical device.

Optimization was carried out using Whatman filter paper no. 1, 42, 6, CF 1, CF 3, and 1 CHR cut to a size of 0.7 × 0.7 cm. In total, 20 µL of the extract solution at a certain pH based on the previous stage was dripped on the papers. Papers containing betalain were then dried at room temperature until all the solvent evaporated. Next, 10 µL of Cu and Fe solutions was added to the paper dripwise at 25 mg/mL. This method was adapted from Pratiwi et al. (2019), with adjustments made according to the stability of the betalain pigments^34^. Paper containing betalain was attached to the end of an inert plastic material measuring 0.8 × 4 cm as a support for the PAD (Fig. 1).

The extract concentration was optimized by using paper containing betalain at a lower concentration. Extract solutions were created with 500, 400, 300, 200, and 100 mg/mL and 20 µL of each solution dripped onto the papers. Cu and Fe solutions from the previous step were diluted 12.5 times to 2 mg/mL, and 10 µL of each solution was dripped onto the paper. The metal concentration was optimized by developing 10 solutions of each ranging from 0 to 200 mg/L, with 0 mg/L representing demineralized water without the addition of metal. We then recorded the color change at the lowest metal concentration that could still be observed visually.

#### Sensitivity and performance test of betalain PAD

The sensitivity test was carried out using six concentrations ranging from 0 to 5 mg/L of Cu and Fe. In total, 10 µL of each solution was dripped onto the PAD, and the color change was observed. Quantitative analysis was carried out using the ImageJ software, which aimed to interpret color intensity based on proportional metal content. The photo was taken at a distance of 10–15 cm vertically from the position of the paper, without a flashlight, using an even brightness level of 20%. The analysis settings in ImageJ included hue 0, saturation 60 (Cu) and 70 (Fe), and brightness 0. All pictures were inverted.

PAD was also tested on drinking water, tap water, and well water using the standard addition method^16^. The samples were collected from residential areas in Bandung, Indonesia. Spiked samples were generated by adding Cu and Fe separately, with quantities based on the EPA’s allowable levels^6^. To digest the samples, 5 mL of 65% HNO3 was added and boiled until reduced to 10–15 mL. Then, demineralized water was added until reaching a total quantity of 100 mL (pH < 2). The samples were then dropped individually onto the PAD. Color variations within 60 min were recorded and compared to standard colors for quantitative analysis.

### Betalain complex ratio estimation with job’s plot

The Job’s plot method was used to predict the mole ratio of Cu or Fe to betalain in the complex compounds. The estimation was performed by adjusting the volume of extract and metal solutions with the same concentration. Three separate solutions were initially prepared: (1) an extract solution with betanin content equal to 30 mM 2-decarboxy betanin, (2) a 30 mM Cu metal solution, and (3) a 3 mM Fe metal solution. The extract and each metal solution were mixed with a volume ratio of 5:0, 4.5:0.5, 4:1, 3.5:1.5, 3:2, 2.5:2.5, 2:3, 1.5:3.5, 1:4, 0.5:4.5, and 0:5. All total volumes were equal. Cu and Fe were added to the extract solution with the specified volume variations, and absorbance was measured at the λmax to determine the color change. The Job’s Plot method was also repeated three times.

## Results and discussion

### Betalain extraction from red dragon fruit

Red dragon fruits (H*ylocereus polyrhizus* (F.A.C. Weber) Britton and Rose) featured red–purple skin and flesh with dense black seeds, with a fruit diameter of 11 cm and a peel size of 1 cm. From 1.37 kg of fruit, 53.9 g of flesh extract was obtained, equivalent to 5.44% yield per 100 g of fresh flesh. Using the same extraction method, Rodriguez et al. (2015) reported that the betalain content in red dragon fruit was 42.71 + 2.48 mg per 100 g of fresh fruit^29^. Betalain is a pigment that dissolves in polar solvents; nonetheless, an ethanol solvent is preferred over water for extracting betalain due to the relatively low extraction efficiency and stability of betalain in aqueous solvents^35,36^. The addition of citric acid to betalain extracts is known to increase the levels of extracted betalain^37^. However, compared to the use of a water–citric acid solvent alone, extraction with ethanol can produce a greater yield since the water–citric acid solvent only extracts 90% of the betalain extracted using an ethanol solvent^35^.

### Betalain characterization

#### Qualitative analysis with thin layer chromatography

**Fig. 2:**
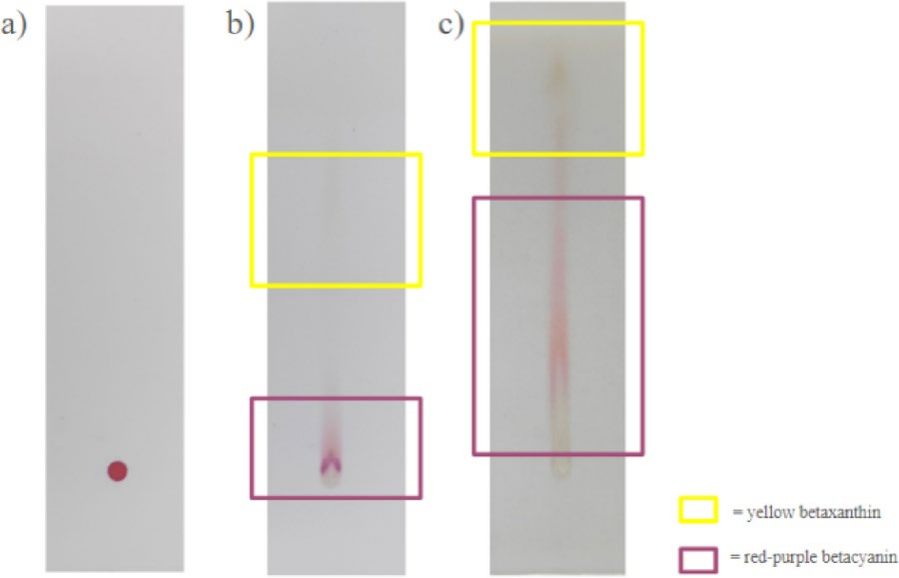
Thin layer chromatography of red dragon fruit **(a)** before elution; **(b)** after first elution with 2-propanol–EtOH–water–acetic acid (6:7:6:1); and **(c)** after second elution with 2-propanol–EtOH–water–acetic acid (11:4:4:1), adapted from Rodriguez et al. (2016)^40^.

A qualitative analysis was performed to demonstrate that the betalain pigment obtained from the extract was a mixture of two colors, red–purple and yellow, derived from betacyanin and betaxanthin. The yellow spot in Fig. 2 shows the presence of betalains from the betaxanthin sub-group, whereas the red–purple spot suggests the presence betalains from the betacyanin sub-group. These findings are consistent with those of previous research, indicating that when betalain extract is exposed to direct light, it produces red–purple and yellow spots^38^. According to Viloria-Matos et al. (2001), by using HPTLC with this eluent system, the red betalain pigment appears at Rf 0.55 as the predominant fraction, indicating a higher content, whereas the yellow pigment appears at Rf 0.72. The different levels of color dominance between the red–purple and yellow spots may occur due to different types of red dragon fruit, which produce distinct betalain derivatives. Several betalain derivatives in red dragon fruit have been identified, including betanin, isobetanin, phyllocactin, hylocerenin, isophyllocactin, and isohylocerenin^39^. A similar eluent technique can also be used to purify betalains by separating betacyanin and betaxanthin. Cellulose plates are advised to improve separation^40^.

#### Qualitative analysis with UV-Visible spectrophotometry

UV-visible spectrophotometry was used to confirm the presence of betalain pigment in dragon fruit. This analysis was carried out in the visible light region (320–900 nm). The result shows that betalain has maximum absorbance at 532–533 nm for red–purple betacyanin (530–540 nm)^33^. The maximum absorbance of betacyanin pigment was determined to occur at 537 nm^41^. A betacyanin derivative with a maximum wavelength of 533 nm was reported to come from 2-decarboxy-betanin^42^. Other betacyanin derivatives with a similar λmax include 15-decarboxy-betanin (528 nm), betanin (538 nm), and betanidin (540 nm)^42^.

#### Stability study of betalain extract

The stability study of the betalain extract was carried out at a temperature of 5 *±* 3 °C, with humidity monitoring findings ranging between 38 and 50%. This study was carried out by preparing an extract solution with absorbance of 0.7 (13.6 mg/mL) and a maximum wavelength of 533 nm. Within 16 weeks, the extract’s absorbance decreased by up to 10.6% from week 0. This result demonstrated that the hue of the betalain extract was stable at cold temperatures (5 *±* 3 °C) for at least 16 weeks. Then, the color faded significantly after 16 weeks in storage. This stability test demonstrated that storage at 5 *±* 3 °C can maintain betalain stability. Fading color in betalain is a sign of instability due to degradation in cases where the substance can not maintain its intensity. There are several external factors contributing to color instability such as the matrix effect including the water content of the extract. In this case, water activity possibly induces hydrolytic reactions of betalain derivatives susceptible to aldimine bond cleavage^43^. A preventive strategy can be applied by drying pigments with water content below 10% (at least) and below 5% (optimally) to minimize degradation^44^.

Betanin and isobetanin, betalain derivatives, can be degraded into a variety of colored derivatives, including 2-decarboxy-betanin (red), 17-decarboxy-betanin (red-orange), betanidin (red–purple), 15-decarboxy-betanin (red), neobetanin (yellow), isobetanidin (red–purple), 17-decarboxy-isobetanin (red-orange), and 2-decarboxy-isobetanin (red). Forming degradation products is indicated by wavelength shifts and changes in color intensity. The decarboxy form of betanin causes a hypochromic shift, which is followed by a drop in absorbance. This shift occurs because the yellow–orange tint maximizes absorption at shorter wavelengths. One example is the creation of neobetanin (yellow) after degradation, marked by absorption at a λmax of 470 nm^43^. The bathochromic shift can appear when the red pigment becomes red–purple in the formation of betanidin (540 nm) from betanin (538 nm)^42^. Degradation occurs through reactions such as deglycosylation, hydrolysis, decarboxylation, and dehydrogenation, as well as through a combination of decarboxylation and dehydrogenation. In an acidic environment, exposure to high temperatures and the presence of the β-glucosidase enzyme betanin will lead to deglycosylation, where the glucose group on the betanin molecule is cut off and produces betanidin. In this reaction, the red color (betanin) becomes more intense or changes to red–purple (betanidin)^43^. Hydrolysis of betanin can also occur and cause a reversible reaction that produces betalamic acid (yellow) and cyclo-Dopa-5-O-β-glucoside (colorless). This reaction converts the red betalain pigment to yellow and occurs at pH > 6 with exposure to high temperatures^43,45^. Dehydrogenation produces neobetanin (yellow), which occurs when betanin is exposed to high temperatures and aerobic conditions (oxygen) at the same time^46^.

### Selectivity study of betalain pigment on metal ions

A selectivity analysis was conducted on 12 metal ions to determine which metal reacts with betalain, resulting in a substantial color shift. Acetic acid and ammonium hydroxide were added to the 13 mg/mL extract solution (pH 4–5) to produce a pH range of 2–11. Meanwhile, the pH 4 variation required no acid to modify the pH. Then, 30 mM metals (Na, K, Ca, Ba, Al, Mg, Zn, Fe, Cu, Hg, Ni, and Pb) were added to each solution at a metal–betalain volume ratio of 1:1. Sediment-containing samples were spun at 5000 rpm for 5 min in a centrifuge tube.

**Fig. 3:**
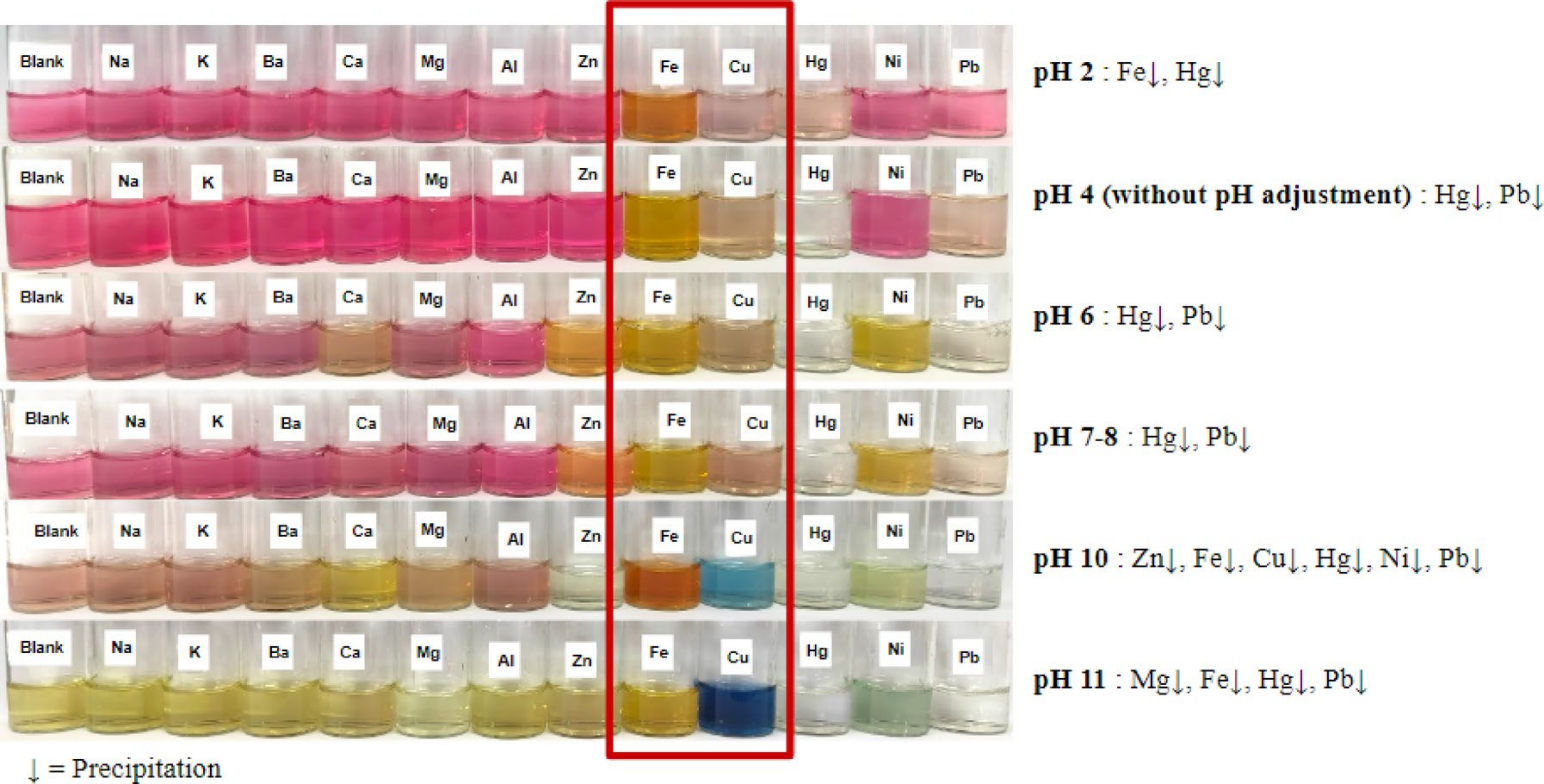
Selectivity of betalain pigment on metal ions at pH 2–11 with adjustments using acetic acid and ammonium hydroxide.

**Fig. 4:**
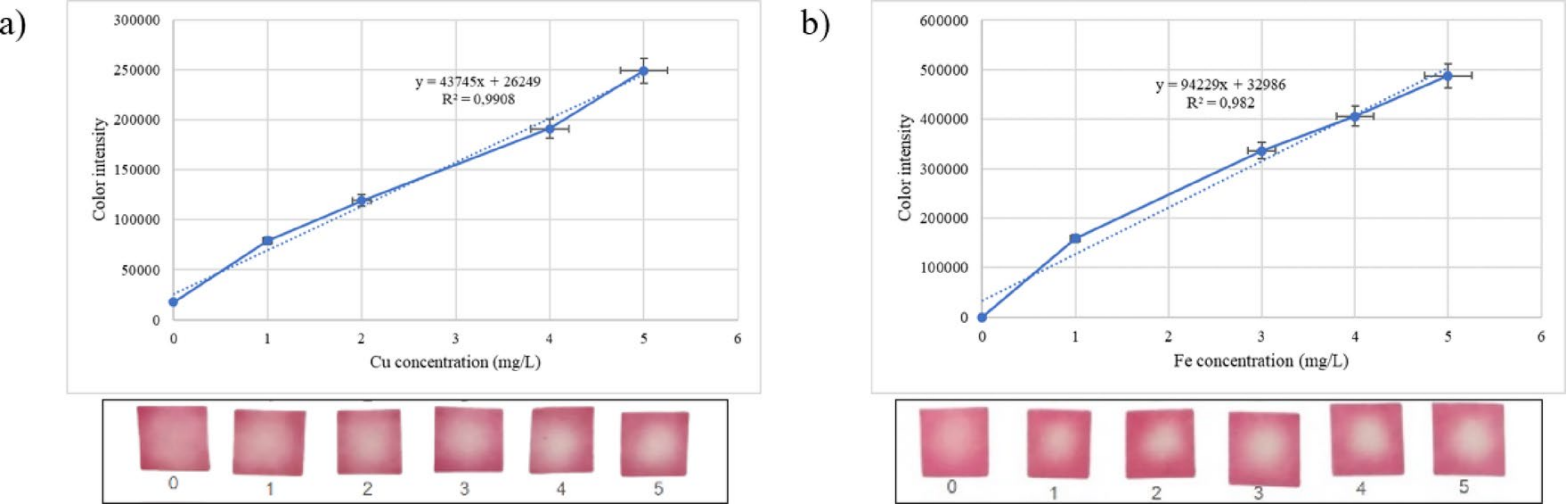
Job’s plot curve for estimating the complex ratio of **(a)** betalain–Cu and **(b)** betalain–Fe.

The extract solution changed color from pink to yellow with the addition of acid and base in the blank solution of pH 2–11 (Fig. 3**)**, even though no metal was added. This result is consistent with the literature, which indicates that pH affects the color of betalain pigment, with the pink color disappearing dramatically at pH > 8^47^. The metals Fe and Cu consistently induced color changes at all pH variations when compared to eleven other metals, including the extract at pH 4, which was not supplemented with an acid or base. In the extract solution at pH 4, Fe changed the pigment color from pink to yellow, whereas Cu metal changed the pigment color from pink to pale orange. Additionally, adding metal to the extract at pH 4 caused a shift in the λmax value. Conversely, Pb and Hg yielded considerably lower absorbance in the visible region, leading the solution to appear colorless (Fig. 4). Fe and Cu altered the spectra dramatically by increasing absorption at other wavelengths. The addition of Cu caused a change in λmax from 533 nm to 500 nm and the development of a new λmax at 797 nm. The addition of Fe resulted in absorbance > 1.00 at a wavelength < 400 nm. Polar solvents can cause color deterioration by shifting the λmax to a shorter wavelength (hypochromic) and producing degradation products at 430 nm^23^. In this situation, it is expected that the same decomposition will occur due to the close polarity of the polar solvent utilized, followed by metal exposure to the betalain extract, leading to a hypochromic shift.

According to Guerrero-Rubio et al. (2020), the change in absorbance after adding Cu2 + to betanin occurs due to the creation of a complex between the pigment and metal, rather than a pigment breakdown caused by metals. This result is corroborated by the presence of a reversible effect when the Cu–betanin mixture is combined with tropolone as a Cu chelator, indicating that Cu–tropolone has a higher affinity than Cu–betanin. A shift in λmax was observed from 537 nm (betanin) to 508 nm (Cu–betanin)^48^.

The color altering impact of betanin due to metal exposure was also observed with the addition of Fe metal via complex formation, resulting in degradation of the pigment color. Compared to Cu, the color shift when adding Fe was considered modest^49^. Metal cations can attach to betalains by complexing with the 2,6-dicarboxyl-1,2,3,4-tetrahydropyridine group of the betalamic acid component of the betalain group molecule^50^. This stage led to the selection of Cu and Fe metals for use in the development of PAD. Cu and Fe also showed the most significant color change at pH 11 and 10, respectively, compared to the blank solutions of each pH variation (Fig. 3**)**. However, pH adjustments on portable devices like PAD will require additional reagents in the future, increasing the process’s complexity due to the inevitable use of a liquid reagent. Therefore, pH 4 (without acid/base adjustment) is preferred. The pH 4 condition was also chosen since the color change was easily noticeable even without acidic/basic modifications.

### Design and optimization of betalain paper analytical device

Paper types were optimized on six different papers: Whatman 1, 42, 6, CF 1, CF 3, and 1 CHR, each with a different thickness and pore size. Whatman paper no. 1 has a thickness of 180 μm and a pore size of 11 μm. Whatman ashless paper no. 42 has a thickness of 200 μm and a pore size of 2.5 μm. Whatman paper no. 6 has a thickness of 180 μm and a pore size of 3 μm. These three papers are most typically used for filtration, for which a smaller pore size enables more particles to be filtered at a slower rate^51^. The CF 1 paper has a thickness of 176 μm and a water absorption capacity of 18.7 mg/cm3, while CF 3 has a thickness of 322 μm with a water absorption capacity of 34.6 mg/cm3. Both papers are commonly used in the development of colorimetric methods that require observations through the lateral flow of liquids in small volumes^52^. The thickness of CF3 paper is large enough to absorb more reagents than other types of paper. Type 1 CHR paper (180 μm thickness) is often employed in paper chromatography procedures, where the speed of liquid flow considerably effects the separation of a mixed sample (Evans et al., 2014). To optimize the paper, 20 µL of 1000 mg/mL extract was dripped as a reagent blank (Table 1) onto each 0.7 × 0.7 cm piece of paper.

**Table 1:**
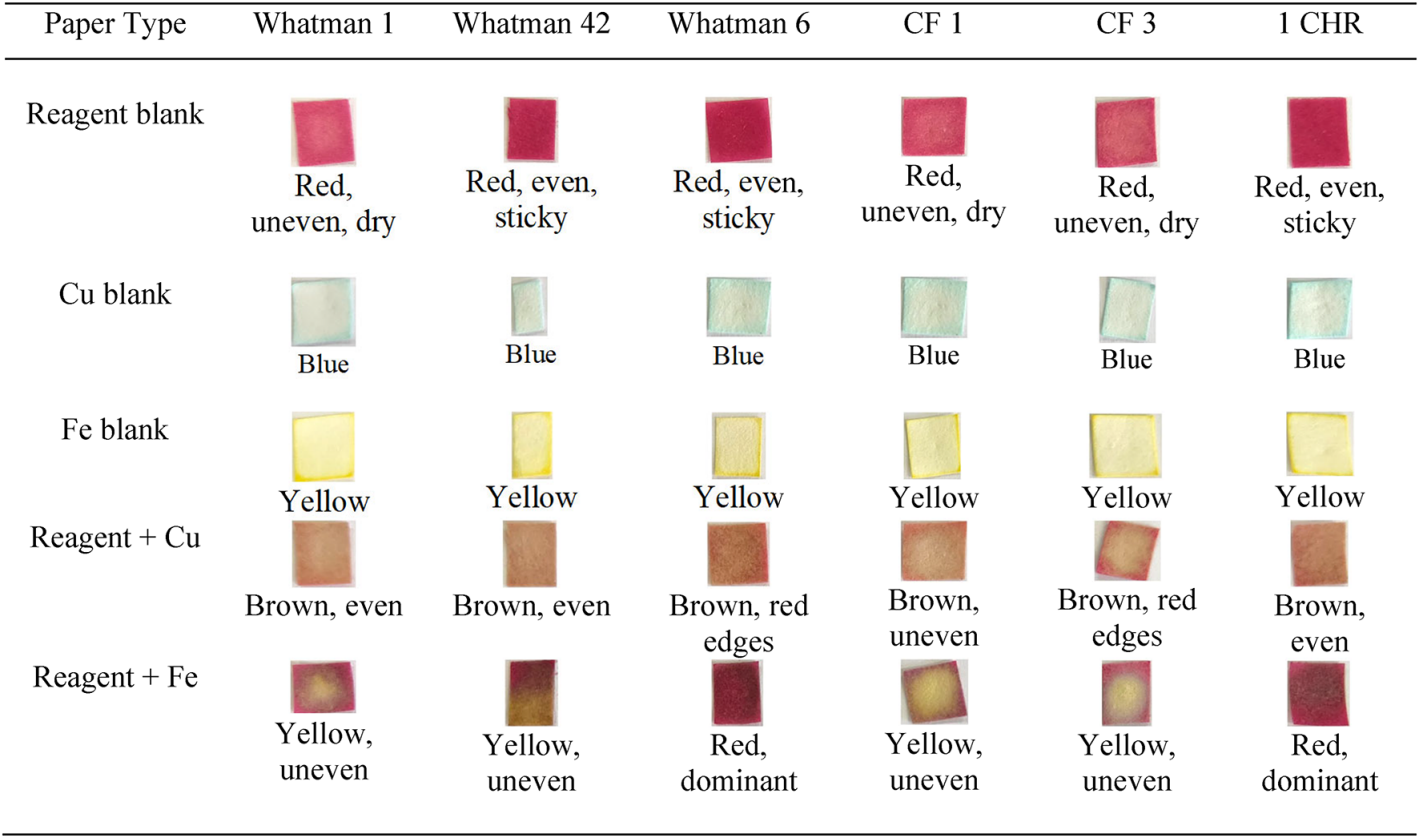
Optimization of paper type, phase 1.

Table 1 shows that reagent blanks on Whatman 1, CF 1, and CF 3 paper generated an uneven color on the dry surface. However, Whatman 42, 6, and 1 CHR produced sticky paper. Paper with larger pore sizes and thicknesses can absorb chemicals more quickly, thereby improving drying. Porosity is a key element in fluid absorption. A more porous shape promotes increased liquid absorption because there is more space for water molecules to interact with paper fibers^53^. Whatman 42 and 6 paper have smaller pore sizes. Therefore, reagent absorption is slower, and the paper’s surface becomes moister and stickier.

After drying for 30–45 min, 10 µL of Cu 0.75 mg/µL or Fe 1.6 mg/mL solution was dropped onto each paper. The presence of Cu and Fe metals caused more obvious color changes, including brown and yellow on the Whatman 1, CF1, and CF3 paper. The Whatman 1, CF 1, and CF 3 paper were tested again at a lower extract concentration of 500 mg/mL, with the addition of 10 µL of Cu and Fe at 25 mg/mL.

**Table 2:**
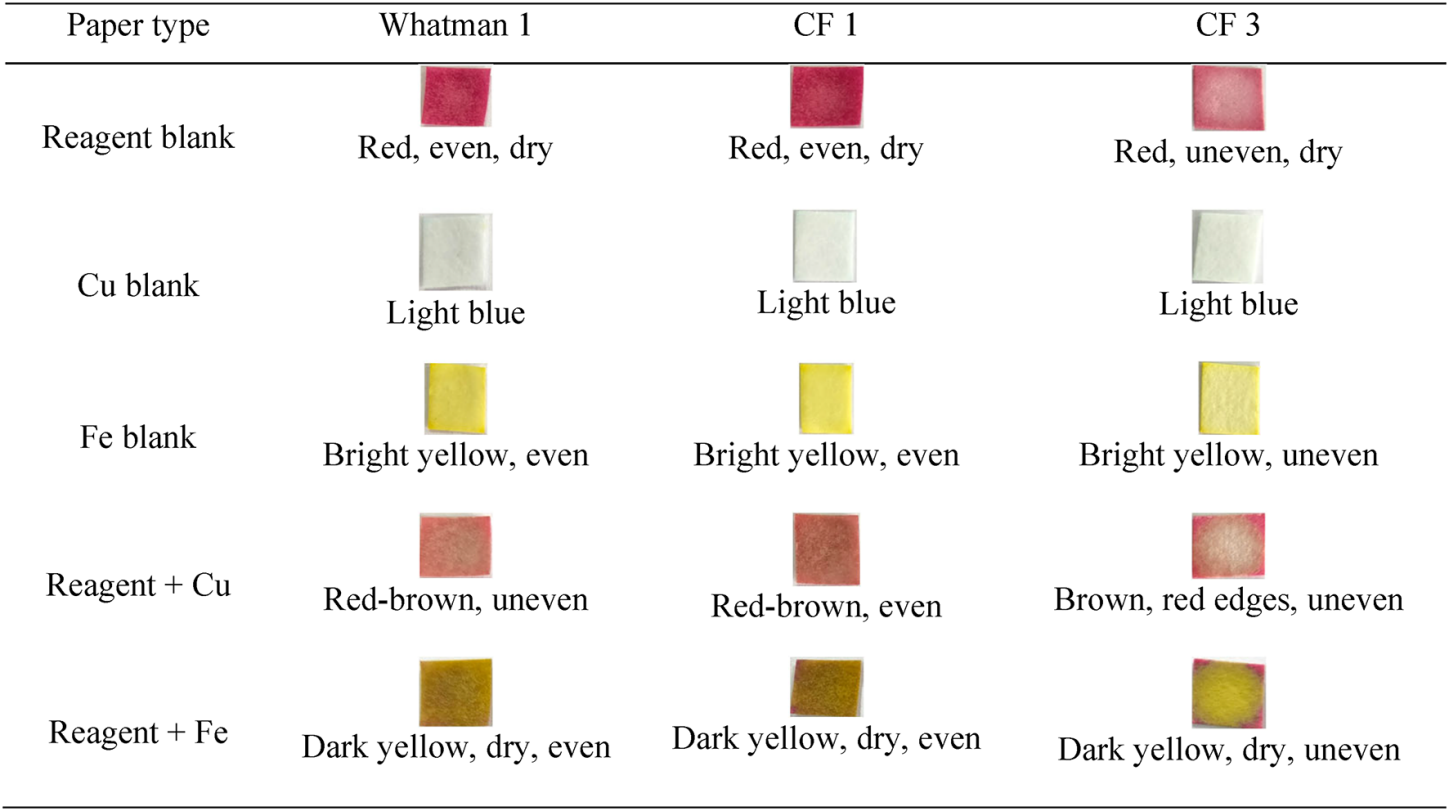
Optimization of paper types, phase 2.

At lower concentrations (Table 2), the reagent blank on CF 3 paper became faded, although Whatman 1 and CF 1 remained concentrated. This phenomenon is connected to the thickness of the CF 3 paper, which can absorb more reagents. The more the reagent becomes absorbed into the paper, the more concentrated the metal required to achieve a distinct color shift becomes^54^. In the drip findings (reagent + Cu and reagent + Fe), the CF 1 paper produced a slightly more uniform color than Whatman 1. Based on the results of the two paper optimization methods, the CF 1 paper was chosen because it has the same metal concentration (25 mg/mL).

### Optimization of extract and metal concentrations

The extract concentration was optimized to establish how much reagent was required to generate betalain paper with a bright color while detecting metal at extremely low levels. This process was performed to achieve PAD with great sensitivity. To optimize the extract concentration, we used various dilutions of 500, 400, 300, 200, and 100 mg/mL, with 20 µL of each solution dripped over CF 1 paper. The Cu and Fe metal concentrations were diluted 12.5 times to 2 mg/mL and dripped in a quantity up to 10 µL.

**Table 3:**
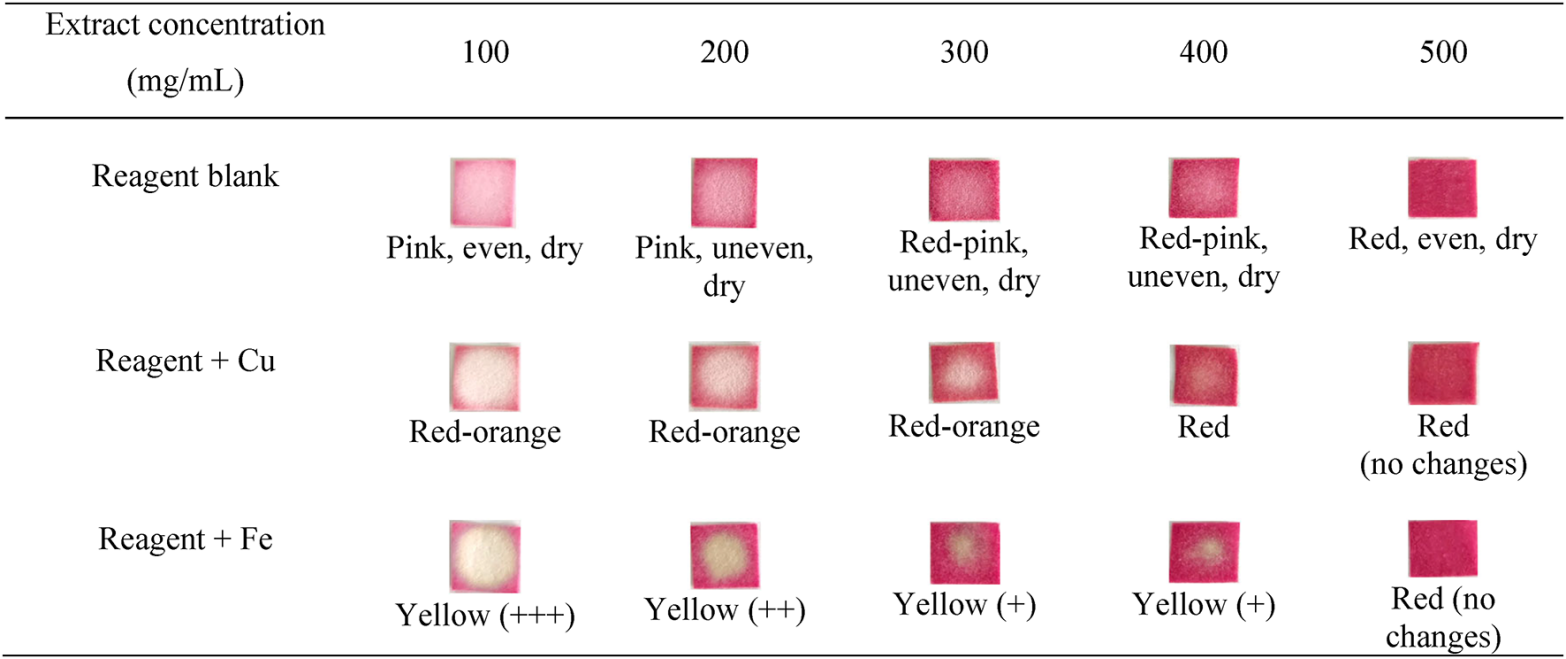
Optimization of the extract concentration.

As shown in Table 3, the paper containing the 100 mg/mL extract yielded an even, dry pink color with an obvious color change. The color change after adding Cu and Fe occurred primarily in the middle area around the drop point. The 100 mg/mL extract concentration was chosen based on the color shift observed when the metal was added.

To optimize the metal concentrations, the solution was diluted into 10 variations ranging from 0 to 200 mg/L, each with a drop volume of 10 µL. Visually, the color changes from 200 to 0 mg/L appeared gradational, with subtle color variances between concentrations. Cu and Fe samples could be seen under visible light at concentrations above 100 mg/L. However, at values below 100 mg/L, the color shift was difficult to detect. Quantitative analysis at a lower level (< 10 mg/L) was continued using ImageJ.

### Sensitivity and performance test of the betalain paper analytical device

Quantitative testing is required to determine the sensitivity of PAD and achieve metal detection at low levels. For this purpose, we used the ImageJ software, which interprets color intensity proportionately to metal content. The sensitivity test was performed by preparing six concentrations ranging from 0 to 5 mg/L and then dripping each solution onto betalain paper under optimal conditions. Color variations were captured with a cell phone camera and analyzed via ImageJ. The photograph was taken at a distance of 10–15 cm vertically from the paper’s position using an even brightness level of 20% without a flashlight. The detected color intensity was then plotted to produce a linearity curve.

**Fig. 5:**
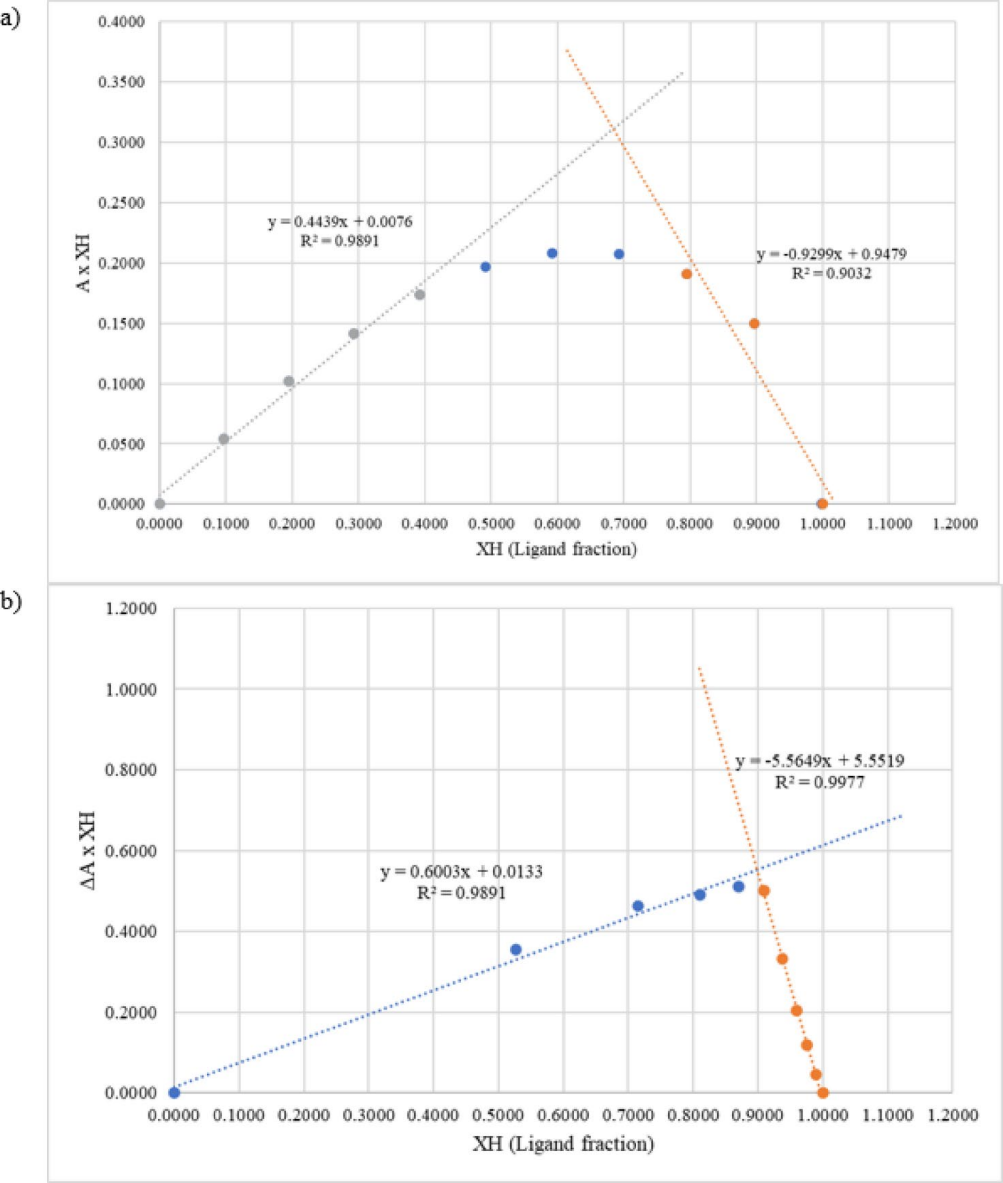
Linearity curve and visual appearance: (**a**) Cu and (**b**) Fe.

According to the linearity curve (Fig. 5), the quantification limits for Cu and Fe metals were 3.133 mg/L and 4.736 mg/L, respectively, while the detection limits were 1.034 mg/L and 1.563 mg/L. Betalain PADs were also applied for drinking water, tap water, and well water. Cu and Fe were added to the spiked samples at concentrations of 1.3 mg/L (Cu) and 0.3 mg/L (Fe), respectively, in accordance with the EPA’s maximum metal limit standards for water. The three water samples were then digested with HNO3 and placed on the papers for analysis with imageJ. The test findings are summarized in Table 4.

**Table 4:** Determination of Cu and Fe using betalain PAD in the water samples.

Samples	Spiked Cu Content (mg/L)			Spiked Fe Content (mg/L)		
	Betalain PAD		AAS Instrument	Betalain PAD		AAS Instrument
	Non-digested	Digested	Digested	Non-digested	Digested	Digested
Drinking water	1.659±1.16	1.369±0.44	1.383±0.007	0.506±0.04	0.521±0.03	0.201±0.009
Tap water	1.573±1.33	1.889±0.46	1.375±0.006	0.512±0.06	0.440±0,23	0.345±0.008
Well water	2.092±0.77	2.272±0.63	1.358±0.021	0.478±0.02	0.719±0.10	0.498±0.027

Sensitivity tests on real samples showed varying values of Cu and Fe levels. Based on the test using the AAS, the spiked samples yielded Cu levels of 1.3825, 1.375, and 1.3575 mg/L, respectively, for drinking water, tap water, and well water. Compared to the AAS results, the samples of drinking water, tap water, and well water without digestion yielded percent deviations of 20%, 14%, 54%, respectively. With digestion, these values were 1%, 37%, 67%, respectively. The Fe test findings show that the samples had iron concentrations of < 1 mg/L, which remains lower than the indicator strip detection limit of 1.563 mg/L, even after the addition of 0.3 mg/L Fe.

Various factors affect the sensitivity of PAD, e.g., when one of each is homogenous or the reagent is distributed in the paper as the substrate. A lack of reagent homogeneity in the paper resulted in different measurement areas. The process of drying the paper after dropping the reagent frequently results in paper that is darker on the edges than in the center. This phenomenon, also known as the coffee-ring effect, is generated by the higher rate of solvent evaporation in the edge area, such that the reagent is more dispersed on the edges than in the middle area^55^. As a result, papers with unequal reagents create variations in strip area and color intensity, producing larger metal concentrations than AAS. To achieve a linear area and color intensity relative to the metal content, paper with a uniform reagent composition is required. Studies to improve the color intensity and uniformity in PAD development have reported that paper surface modifications using a printing technique and fluid transport control could increase PAD performance. To reduce the coffee-ring effect, stacking reagent-loaded paper with a fluid-distributor layer allows vertical fluid transport that reduces lateral elution and, therefore, increases uniformity^54^. When using ImageJ, the measurement area is a key factor in determining the levels. In this study, the measurement area was determined as a circle with dimensions of 1976 px2 (Cu) and 1264 px2 (Fe) at the center of the PAD. This point was taken based on a series of test results that showed color changes in the middle area.

The PAD’s performance was evaluated using stability tests at a temperature of 5 *±* 3 °C and humidity levels ranging from 40 to 45%. The paper was stored in a plastic zip lock bag to prevent contamination. We dripped 10 µL of 100 mg/L Cu and Fe metal solutions in triplicate onto to the paper to test its stability based on the optimization and visual observation results. Paper containing betalain showed the same color change reaction even after 55 days of storage. The color change in the Fe samples could be seen immediately after dripping. However, the colour change in the Cu samples required 10–15 min, and the resulting orange color was more visible after the paper was completely dry.

**Table 5:** Comparison of several existing natural pigment-based paper analytical devices for the analysis of Fe and Cu.

Method	Analyte/Sample	Pigment/Natural Source	Substrate	Analytical Approach	Limit of Detection /Limit of Quantification	Refs.
Betalain PAD	Cu in water	Betalain from red dragon fruit (*Hylocereus polyrhizus*)	Whatman CF 1	Smartphone camera and ImageJ	LOD: 1.034 mg/L LOQ: 3.133 mg/L (linearity curve estimation)	This study
	Fe in water	Betalain from red dragon fruit (*Hylocereus polyrhizus*)	Whatman CF 1	Smartphone camera and ImageJ	LOD: 1.563 mg/L LOQ: 4.736 mg/L (linearity curve estimation)	This study
Electrospun zein membrane	Fe in water	Curcumin powder	Zein polymer membrane	Inductively coupled plasma optical emission spectrometry	LOD: 0.4 mg/L (visual observation)	^15^
Capillary-driven microfluidic paper	Fe in water	Anthocyanin from butterfly pea (*Clitoria ternatea*)	Whatman grade 1	Smartphone app (MATLAB) and ImageJ	LOD: 43 mg/L using imageJ 55 mg/L using smartphone app	^56^
Metallochromic cellulose dipstick	Fe in water	Cyanidin from red cabbage	Chitosan nanoparticle cellulose paper strip	Hunter Lab Ultra Scan Pro Spectrophotometer	LOD: 10–400 mg/L (visual observation)	^57^
Bio-film colorimetric sensor	Fe in water	Curcumin powder	Aloe vera–banana starch bio-films	UV-Visible Spectrophotometer	LOD: 27.84 mg/L LOQ: 92.81 mg/L (linearity curve estimation)	^58^

Several studies reported using natural pigments as colorimetric reagents in developing portable devices, as shown in Table 5. Among the five methods above, the electrospun curcumin–zein membrane method yielded a lower detection limit than betalain PAD, which is the closest to the EPA requirement limit. Based on a comparison of the analytical approach, three methods may require trained personnel for quantitative analysis since spectrophotometers are still employed. A combination of smartphones and software such as imageJ and MATLAB is likely to be the most portable and applicable, as such an approach would enable on-site use. Although MATLAB offered a higher detection limit than imageJ^56^, MATLAB could be further considered to support one-device use in future research since this software is available on smartphones while imageJ is only available for computers. Compared to these techniques, our approach of using betalain PAD has potential for on-site analysis without requiring complicated procedures by trained personnel.

### Betalain complex ratio estimation with job’s plot

Formation of betalain–Cu and betalain–Fe complexes were estimated using the Job’s Plot method, which involved a series of mixed solutions containing the extract and metals. The series consisted of a volume ratio of 5:0, 4.5:0.5, 4:1, 3.5:1.5, 3:2, 2.5:2.5, 2:3, 1.5:3.5, 1:4, 0.5:4.5, and 0:5. Each mixture was examined using a UV-Vis spectrophotometer. The absorbance of the betalain–Cu complex was measured at 502 nm, whereas betalain–Fe was measured at 533 nm. The absorbance value of the mixtures and molar ratio of 2-decarboxy betanin, Cu, and Fe were plotted on a graph to determine the intersection point of the two curves. This point represents the optimum molar ratio of betalain and Cu or Fe that to form a complex compound.

In Fig. 4**(a)**, the betalain and Cu fraction curves intersect at x = 0.689, which represents a metal–ligand ratio of 1:2. The formation of a Cu–betalain complex with a ratio of 1:2 can occur by forming bonds on the N atom of the tetrahydropyridine group and two O atoms of the two carboxylic groups^59^. Meanwhile, in Fig. 4**(b)**, the betalain and Fe fraction curves intersect at x = 0.898, corresponding to a metal–ligand ratio of 1:9. The Job’s plot curve indicates a metal–ligand ratio of 1:1 when the two curves intersect at x = 0.5 or have a ligand fraction = 0.5. The intersection at x = 0.67 represents a ratio of 1:2^60^.

According to our research findings, the dripping technique adopted in this study had an unfavorable effect on the reagent distribution of the papers. This technique caused a coffee ring effect, and, as a result, the betalain PAD results differed from those of the established method, representing a limitation of this study. Although this study was less sensitive to detecting very low amounts, it did offer a quick color change and could be a feasible alternative for on-site detection of Cu and Fe in samples.

## Conclusions

In this study, a betalain paper analytical device was developed using Whatman CF 1 paper and 20 µL of 100 mg/mL betalain extract at pH 4–5. No acids or bases were added. The betalain PAD changed color from pink to pale orange under the Cu analysis and from pink to yellow under the Fe analysis. The device’s quantification limit was 3.133 mg/L (Cu) and 4.736 mg/L (Fe), while the detection limit was 1.034 mg/L (Cu) and 1.563 mg/L (Fe). The estimated ratios for complex formation are 1:2 (Cu–betalain) and 1:9 (Fe–betalain). According to the stability study, the betalain extract should be stored at 5 *±* 3 °C. Based on the present results, more research into betalain PAD is needed, with an emphasis on increasing sensitivity. For further improvements, we propose using an alternative approach to fabrication that introduces the betalain reagent to the paper substrate via printing techniques and the implementation of strategies for fluid transport control.

## Data Availability

Data is provided within the manuscript.
